# Cervical cancer trend in the Republic of Kazakhstan and attitudes towards cervical cancer screening in urban and rural areas

**DOI:** 10.1038/s41598-024-64566-8

**Published:** 2024-06-14

**Authors:** Indira Zhetpisbayeva, Alexander Rommel, Fatima Kassymbekova, Yuliya Semenova, Sholpan Sarmuldayeva, Azhar Giniyat, Gulnaz Tanatarova, Azhar Dyussupova, Raida Faizova, Venera Rakhmetova, Natalya Glushkova

**Affiliations:** 1 Department of General Medical Practice-2, Asfendiyarov Kazakh National Medical University, St.Tole Bi 94, 050000 Almaty, Kazakhstan; 2 Department of Public Health and Social Sciences, Kazakhstan’s Medical University “Kazakhstan School of Public Health”, St.Utepova 19a, 050060 Almaty, Kazakhstan; 3 Department for Epidemiology and Health Monitoring, Robert Koch Institute, Berlin, Germany; 4 School of Medicine, Nazarbayev University, Astana, Kazakhstan; 5 Department of the International Medical Faculty, University of International Business, Almaty, Kazakhstan; 6 NJSC “National Center for Children’s Rehabilitation”, Astana, Kazakhstan; 7 Department of Healthcare of Abay Region, Semey, Kazakhstan; 8 Department of General Medical Practice, Semey Medical University, Semey, Kazakhstan; 9 Department of Internal Diseases, Astana Medical University, Astana, Kazakhstan; 10 Department of Epidemiology, Evidence Medicine and Biostatistics, Al-Farabi Kazakh National University, Almaty, Kazakhstan

**Keywords:** Health care, Medical research, Oncology

## Abstract

Cervical cancer (CC) continues to be a significant global health issue, which in part can be attributed to disparities in access to CC screening services. This study aims to conduct a trend of CC in Kazakhstan and to compare attitudes towards the screening program between women living in urban and rural areas. In the first stage, we conducted a trend study of CC indicators in Kazakhstan using official statistics. In the second stage, a cross-sectional study was conducted using a structured questionnaire to assess adherence to screening. The trend study reveals a decline in cervical cancer mortality rates (from 7.15 to 5.93 per 100,000 female inhabitants) over the period studied, while the incidence remains stable (from 18.51 to 19.38 per 100,000 female inhabitants). Regional variations in Period Prevalence rates were observed. Significant differences were found in screening participation rates between urban n = 41 (74%) and rural n = 23 (38%) women, *p* < 0.001, as well as awareness of the screening program (urban: n = 15 (27%), rural: n = 35 (58%), *p* < 0.001). The trend study highlights a decrease in cervical cancer mortality rates over the specified period, accompanied by a consistent incidence rate. Additionally, regional disparities in period prevalence rates of cervical cancer were observed. The primary factor contributing to the low adherence of rural women to screening was found to be a lack of awareness regarding the screening program. Therefore, increasing awareness about the importance of screening is crucial for improving adherence rates among rural women in Kazakhstan.

## Introduction

Cervical cancer (CC), caused by persistent infection with high-risk HPV types, is a major public health issue and one of the most common cancers affecting women globally. According to the International Agency for Cancer Research (IARC), it is the fifth most common cancer worldwide^1^. In Kazakhstan, CC is the second most common cancer in women after breast cancer and ranks 10th in mortality among all cancers. The highest mortality rate occurs between ages 30 and 54, coinciding with the most socially active period of life^2^. The estimated age-standardized rates (ASRs World standard) of cervical cancer incidence are relatively low in Western Europe (7.3 per 100,000) and North America (6.6 per 100,000). In contrast, Central Asian countries, including Kazakhstan, have high rates (19.3 per 100,000)^3^. The incidence is 15% lower in urban areas compared to rural areas, with a more significant decline in urban regions (10.2% versus 4.8%)^4^. The WHO emphasizes the need for screening programs to detect precancerous and preclinical forms of cervical cancer due to its increasing prevalence^5^.

Kazakhstan has two cervical cancer screening programs: a national population-based screening for women aged 30–70 and opportunistic screening for older women visiting gynecologists. Pap smear screening, using the Bethesda system since 2011 and liquid-based cytology since 2014, covers 45.9% of the target population^6,7^. Screenings, conducted every four years, are free for women aged 30–70, with invitations primarily via telephone^8^. Positive results lead to free medical care and therapy. Common methods include Pap smears and HPV DNA testing. Accessible screening programs are crucial, especially in low to middle-income countries. Implementing HPV vaccination could significantly reduce cervical cancer incidence, though it is currently not available in Kazakhstan^6–11^.

Risk factors for CC include early sexual activity, multiple sexual partners, immunodeficiency, smoking, and long-term oral contraceptive use. In East Kazakhstan, additional risk factors include lower education levels, later onset of menstruation, cervical manipulations, and any form of contraception. In young women in Almaty, early sexual activity, smoking, and hormonal contraceptive use are common risk factors. In Kazakhstan, HPV types 16, 18, and 31 are particularly prevalent among women aged 18–25 compared to those aged 25–30^12–15^. Although mortality from cervical cancer has been reduced by 50–75% in countries with high participation rates in preventive screening programs, the effectiveness of screening programs is achieved only when the coverage of the target population is 70% or higher^16^. However, in the Republic of Kazakhstan (hereafter—Kazakhstan), the coverage of the target population for cervical cancer screening is only 48–50%^16–18^.

The objective of this study is to investigate the trend of cervical cancer in Kazakhstan and to compare the attitudes towards the screening program among women aged 30 to 70 years between urban and rural areas as the target age group for cervical cancer screening^8^.

## Materials and methods

### Trend study of CC in Kazakhstan

This retrospective study used data sourced from the Kazakhstan Cancer Registry (KCR), which serves as a repository for all histologically confirmed cancer cases in the country and is managed by the Center for E-Health and Kazakh Institute of Oncology and Radiology (KazIOR). To comply with standard practice, healthcare facilities are required by the Ministry of Health (MoH) to report newly diagnosed cancer cases to regional oncological dispensaries using a special form. The regional oncological dispensaries then manually transfer this data to the electronic KCR.

We conducted a trend study of primary indicators of cancer service, including the calculation of incidence and mortality rates associated with cervical cancer (CC) in Kazakhstan over a period of seven years (2013–2019). The standardization process was carried out using the European Standard Population (ESP) and World Standard Population (WSP)^19^. Additionally, we analyzed the period prevalence of cervical cancer among women aged 30–70 years, the target age group for cervical cancer screening, in different regions of the Republic of Kazakhstan over a ten-year period (2011–2021). In 2021, the population of women in the target age group was 4,109,675 and the number of cases of cervical cancer was 1562 cases. To evaluate change over time, relative change with 95% Confidence Interval (CI) was computed. The calculation of Relative change was carried out in the statistical software package Programs for Epidemiologists in Windows (WinPepi), version 11.36^20^.

Owing to the limited availability of requisite source data for calculating incidence and mortality rates for the Republic as a whole, calculations of these indicators were carried out in the time interval corresponding to the available data (2013–2019).

Period Prevalence indicators calculated in detail by region and age group, the available data in these regions covered the period 2011–2021.

Incidence and mortality rates for cervical cancer were calculated per 100,000 female inhabitants using information on the average population size obtained from the Official Statistics website of the Republic of Kazakhstan^21^.

To calculate age-standardized incidence and mortality rates, we multiplied age-specific coefficients per 100,000 population by the age composition standard (ESP or WSP) and summed the products to obtain the final indicator^19^. Furthermore, to compute the prevalence of cervical cancer in different regions of the Republic of Kazakhstan, we standardized the age indicators of each region using the WSP.

### Study on attitude to cervical cancer screening in urban and rural areas

The present pilot study adopted a cross-sectional design and employed a structured and validated questionnaire, adapted from the “Cervical cancer screening adherence” questionnaire^22^, to evaluate the degree of adherence to screening. The survey of respondents was conducted through electronic questionnaire that were distributed via social networks. The subsequent questions have been investigated by place of residence: 1) social characteristics, 2) participation/non-participation and the regularity of screening for cervical cancer, 3) intention to undergo screening, 4) last visit to cervical cancer screening and awareness of the cervical cancer program, 5) reasons for non-attendance of cervical cancer screening program, 6) awareness about the need for screening.

The questions and suggested answers were presented as follows:

*Social characteristics*

- Place of residence: Urban/Rural
- Education: Secondary/Secondary professional/Incomplete higher education/Higher
- Employment status: Employed/Unemployed
- Insurance profile: Insured/Uninsured/Not aware of the profile

*Participation/non-participation and the regularity of screening for cervical cancer*

- Have you participated in cervical cancer screening?/Yes, I participated; No, I did not participate
- Are you regularly screened for cervical cancer?/Yes, I am screened regularly; No, I am not regularly screened.

*Intention to undergo screening*

- Do you intend to be screened in the near future?/No, I don’t intend; I’m not sure; Of course I intend

*Last visit to cervical cancer screening and awareness of the cervical cancer program*

- When was the last time you were screened for cervical cancer?/I never been screened; I have been screened within the last 5 years; I have been screened within the last 10 years; I have been screened more than 10 years have passed since last time
- What do you know about the cervical cancer screening program in the Republic of Kazakhstan?/Almost nothing; General information; Almost all.

*Reasons for non-attendance of cervical cancer screening program*

- Please indicate the reason why you were not screened?/Lack of information about screening; Lack of understanding of the importance of screening; Lack of time; Fear of being screened; Lack of confidence in the screening program.

*Awareness about the need for screening*

- Why do you think you should be screened for cervical cancer?/Timely diagnosis of the disease; Control of one’s health; I don’t see the need for this, it’s all for reporting; multiple-answer “timely detection of the disease and control of my health.”

#### Sample selection and enrollment of respondents

The study sample was recruited through “snowball sampling” in women between 30 and 70 years, who were eligible for screening and had no prior history of cervical cancer^8^.

The sample size was determined in accordance with the recommended minimum sample size for pilot studies. According to this guideline, with a problem probability of 5% and a 95% confidence level, it is established that the minimum sample size for a pilot study should encompass 59 individuals^23^.

Exclusion criteria included women aged below 30 years or above 70 years, and those with a prior diagnosis of cervical cancer. This encompassed women residing in both rural and urban settings, who either had not undergone screening or had not done so in a considerable time period. To compare and evaluate the adherence to cervical cancer screening and the level of screening awareness between these two groups, participants were segregated into two categories based on their place of residency: Group 1 included 55 urban women, while Group 2 consisted of 60 rural women.

The criterion to attribute of women into urban and rural groups was based on their permanent place of residence (urban/rural). The questionnaire included a question allowing the respondents, to self-identify their place of residence as urban or rural.

### Potential ethical concerns

The study received approval from the Local Ethics Committee of Kazakhstan’s Medical University “Kazakhstan School of Public Health” prior to its commencement (Protocol No: IRB-A328 dated 26/12/2022). All methods were performed in accordance with relevant guidelines and regulations. Informed consent was obtained from all participants, who were provided with information about the study and given the option to decline participation. Participants were assured of the anonymity and confidentiality of the study.

### Statistical analysis

In order to analyze the data, we utilized Statistical Package for the Social Sciences (SPSS) software version 20.0 for Windows to perform statistical analysis^24^. Both descriptive and inferential analyses were employed to examine the results. To assess the distribution of continuous variables, we conducted the Shapiro–Wilk test. For nominal variables, absolute numbers and percentages (%) were presented, and between-group comparisons were made using Pearson’s chi-squared criterion. If more than 20% of expected values were less than 5, Fisher’s exact test was used. To determine statistical significance, the critical level of α-error was set at 5%.

## Results

### Trend study of cervical cancer in Kazakhstan

The incidence of CC was at its lowest in 2013, with a rate of 18.51 per 100,000 female inhabitants. However, the highest crude incidence was observed in 2017, with a rate of 19.92 per 100,000 female inhabitants. The standardized incidence rates, calculated using ESP and WSP, were also highest in 2017, with ESP showing a rate of 24.84 per 100,000 female inhabitants, and WSP showing a rate of 17.27 per 100,000 female inhabitants. The subsequent years showed a decline in these indicators. The percentage change in CC incidence was calculated as 0.29% (− 1.00 to 1.60%) for the crude rate, 0.48% (− 1.48 to 2.48%) for ESP, and 0.28% (− 1.36 to 1.95%) for WSP, respectively.

Regarding mortality rates caused by CC across Kazakhstan over six years there was a decrease in this indicator. The crude rate exhibited a relative change of − 3.59% (− 5.65 to − 1.49%), while the respective changes for ESP and WSP were − 4.36% (− 6.68 to − 1.99%) and − 3.25% (− 5.91 to − 0.51%). For crude rates, the highest value was recorded in 2014, with a rate of 7.81 per 100,000 female inhabitants. Similarly, the maximum standardized mortality rate was also observed in 2014, with ESP showing a rate of 10.61 per 100,000 female inhabitants and WSP showing a rate of 6.76 per 100,000 female inhabitants. This indicator showed a decline from 2014 to 2019, reaching its lowest point in 2019, with ESP exhibiting a rate of 7.71 per 100,000 female inhabitants and WSP displaying a rate of 5.08 per 100,000 female inhabitants (Table 1).

**Table 1:** Crude and standardized rates of incidence and mortality of cervical cancer in the Republic of Kazakhstan, 2013–2019.

Years	Incidence			Mortality		
	Crude rate	Standardized rate		Crude rate	Standardized rate_0_	
		ESP*	WSP*		ESP*	WSP*
2013	18.51	22.31	15.79	7.15	9.71	5.92
2014	19.59	24.63	17.08	7.81	10.61	6.76
2015	19.25	24.48	16.88	7.13	9.82	6.15
2016	18.82	23.32	16.32	7.03	9.51	5.97
2017	19.92	24.84	17.27	6.58	8.91	5.71
2018	19.38	24.26	16.79	6.45	8.43	5.56
2019	18.94	23.45	16.27	5.93	7.71	5.08
Relative change (95% confidence interval)	0.29% (− 1.00 to 1.60%)	0.48% (− 1.48 to 2.48%)	0.28% (− 1.36 to 1.95%)	− 3.59% (− 5.65 to − 1.49%)	− 4.36% (− 6.68 to − 1.99%)	− 3.25% (− 5.91 to − 0.51%)

*ESP European Standard Population.

*WSP World Standard Population.

**Figure 1:**
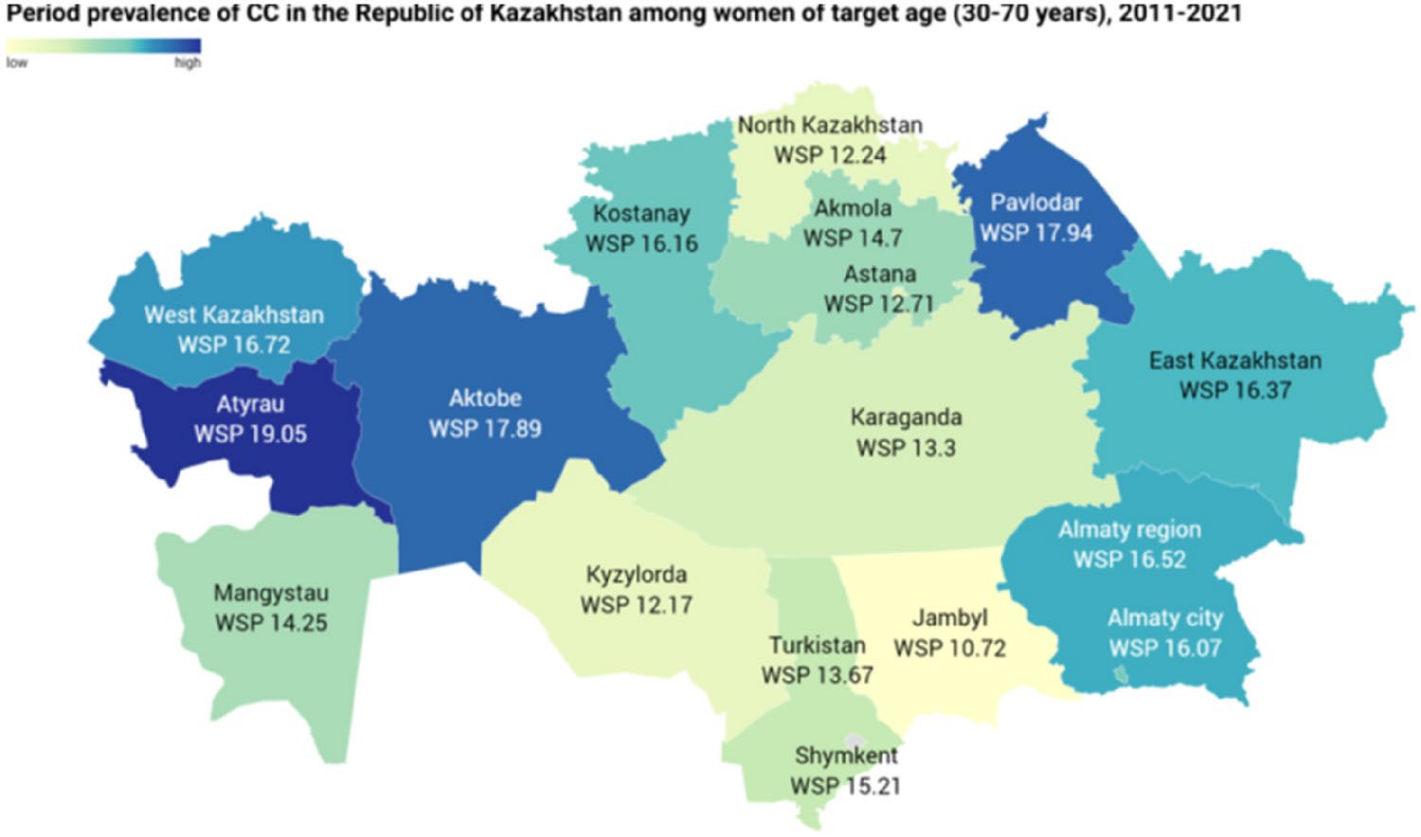
Period prevalence of cervical cancer in the Republic of Kazakhstan among women of target age (30–70 years), 2011–2021.

Figure 1 displays the variations in WSP Period Prevalence across regions. In general, there was a considerable heterogeneity between different regions. The lowest rate was observed in the Zhambyl region, while the highest rate was noted in the Atyrau region. Additionally, elevated rates were observed in the Aktobe, Pavlodar, West Kazakhstan, and Almaty regions.

### Study on attitude to cervical cancer screening in urban and rural areas

Table 2 shows the social characteristic of residents who participated in the study.

**Table 2:** Social characteristics of respondents by place of residence (n = 115).

Characteristic	Place of residence		*P*-value
	Urban (n = 55)	Rural (n = 60)	
Average age	43.36	39.90	
Diff.(95% CI)*	− 3.464 (− 7.44 to 0.44)		
Education*			
Secondary	1(2%)	5 (8%)	0.3807*
Secondary professional	12 (22%)	13 (22%)	
Incomplete higher education	3 (5%)	1 (2%)	
Higher	39 (71%)	41 (68%)	
Employment status*			
Employed	41 (74%)	53 (88%)	0.089*
Unemployed	14 (25%)	7 (12%)	
Insurance profile*			
Insured	42 (76%)	55 (92%)	0.0833*
Uninsured	7 (13%)	3 (5%)	
Not aware of the profile	6 (11%)	2 (3%)	

*Test of difference was Fisher’s exact test.

Diff.(95% CI)—Confidence interval for difference in means.

The average age for urban women was 43.25, for rural women—39.9, the age difference between the two groups was 3.464 (95%CI (− 7.44 to 0.44). A large proportion of women from urban n = 39 (71%), and rural n = 41 (68%) areas had attained higher education. A significant majority of women in both groups were employed, with n = 41 (74%) urban and n = 53 (88%) rural women. The vast majority of participants in both groups had insurance coverage, with n = 42 (76%) urban and n = 55 (92%) rural women. The social characteristics of respondents did not show any statistically significant differences when comparing the two groups.

Table 3 presents a comparison of the frequency of cervical cancer screening participation and non-participation among respondents, categorized by place of residence. Statistical analysis revealed significant differences between the two groups (*p* < 0.001), with rural populations exhibiting significantly lower rates of CC screening participation. Furthermore, a statistically significant difference was observed in the frequency of regular cervical cancer screening participation between respondents living in rural and urban areas (*p* < 0.001). Specifically, female respondents residing in rural areas showed significantly lower regularity in attending cervical cancer screening.

**Table 3:** Participation/non-participation in screening and the regularity of screening for cervical cancer, by place of residence (n = 115).

Characteristics	Place of residence		*P*-value
	Urban (n = 55)	Rural (n = 60)	
*Participation in the screening for cervical cancer*			
Participated	41 (74%)	23 (38%)	< 0.001*
Not participated	14 (25%)	37 (62%)	
*Regularity of the screening for cervical cancer*			
Regular screening	41 (74%)	18 (30%)	< 0.001*
Not screened regularly	14 (25%)	42 (70%)	

*Test of difference was Pearson’s chi square.

**Figure 2:**
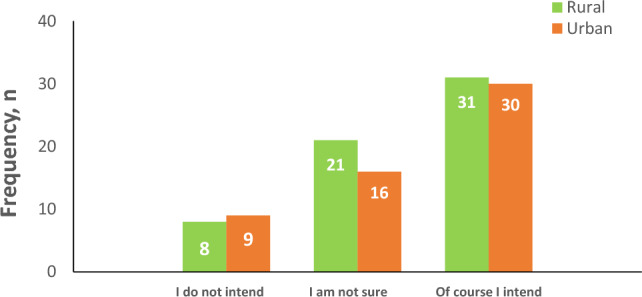
Intention to undergo screening by place of residence.

In the study, one of the analyzed variables was the intention to participate in screening within each group, as presented in Fig. 2. The results indicated that a considerable number of rural women, precisely n = 31 (52%), expressed their intention to participate in the screening program.

Statistically significant differences between the two analyzed groups (*p* < 0.001) were found with respect to the time period in which the last visit to CC screening took place (Table 4). These differences were primarily due to the generally lower participation in the screening program in the rural population.

**Table 4:** Last visit to cervical cancer screening and awareness of the cervical cancer screening program by place of residence.

Characteristics	Place of residence		*P*-value
	Urban	Rural	
*Last visit to cervical cancer screening*			
Never been screened	14 (25%)	41 (68%)	< 0.001*
Within the last 5 years	30 (54%)	17 (28%)	
Within the last 10 years	2 (4%)	1 (2%)	
More than 10 years have passed since last time	9 (16%)	1 (2%)	
*Awareness of cervical cancer screening*			
Almost nothing	15 (27%)	35 (58%)	< 0.001*
General information	25 (42%)	28 (51%)	
Almost all	12 (22%)	1 (2%)	

*Test of difference was Fisher’s exact test.

Table 4 also presents data on the level of knowledge about cervical cancer screening among urban and rural females. Statistically significant results (*p* < 0.001) were identified when comparing screening awareness between the two groups, with the differences attributed to the low awareness of CC screening among the rural female population in Kazakhstan.

**Figure 3:**
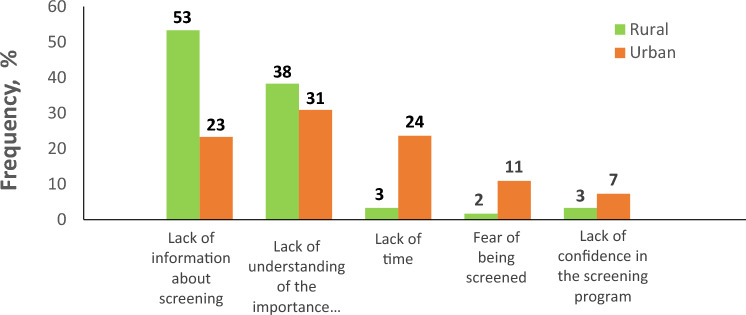
Reasons for non-attendance of cervical cancer screening program by place of residence.

Observing the indicators presented in Fig. 3, it becomes apparent that the rural group exhibits significantly higher rates of “lack of information about screening,” with respondents n = 32 (53%) reporting this as a concern. Furthermore, when comparing the responses on possible reasons for non-participation in the screening between the rural and non-rural groups, statistically significant differences (*p* < 0.001) were found. These differences were largely attributed to the numerous responses from rural women in the target age group who reported a lack of information about screening.

The aim of a further question was to examine the two groups’ awareness about the need for screening. The results showed that rural residents had the highest percentage of respondents n = 40 (67%) in the category of “timely diagnosing,” while one of the most popular options among urban respondents was the multiple-answer option of “timely diagnosis of the disease and control of my health,” with n = 46 (84%) respondents.

Furthermore, the study analyzed the two groups’ opinions regarding the need for a reminder about the screening. The results indicated that the both group of respondents had the high percentage of those who felt the need for reminders about screening: rural residents n = 50 (83%), urban residents n = 42 (76%) respondents. On the other hand, n = 13 (24%) of urban respondents believed that no reminder was necessary, compared to n = 10 (17%) of rural residents. However, no statistically significant differences were identified when comparing the two groups.

## Discussion

### Trend study of cervical cancer in Kazakhstan

The trend study highlights a decrease in cervical cancer mortality rates over the specified period, accompanied by a consistent incidence rate. Furthermore, our study identified regional variations in the period prevalence rates of cervical cancer, with the highest rates observed in the Eastern region and the lowest rates in the Southern region. This variation could potentially be attributed to the differences in the implementation of cervical cancer screening programs across different regions, as well as the territorial and demographic characteristics unique to each region.

According to the Worldwide Cervical Cancer Burden Analysis of 2018, roughly 570.000 women were diagnosed with cervical cancer and 311.000 women lost their lives to the disease. The global age-standardized incidence and mortality rates were 13.1 and 6.9 per 100,000 women, respectively^25^. It is evident that Kazakhstan shows some deviations compared to the global estimates with a higher age-standardized incidence rate and a lower age-standardized mortality rate. A plausible explanation for this discrepancy could be the enhanced implementation of cervical cancer screening programs in the Republic of Kazakhstan, as a result of which the incidence of cervical cancer may have increased.

Another study on the issue of cervical cancer in the Republic of Kazakhstan reported incidence and mortality rates that were consistent with our findings: the incidence rate in this study were 19.5 and mortality rate were 6.4 per 100,000 women^26^.

An earlier study conducted in Kazakhstan revealed an increase in the crude incidence of cervical cancer from 15.24 per 100,000 in 2007 to 18.83 per 100,000 in 2016. The authors attributed this finding to the implementation of a national health program in 2011, which led to improved early detection of cervical cancer through PAP screening. As a result, the same study observed a decreasing trend in cervical cancer mortality, from 7.8 in 2007 to 7.0 in 2016, which was also attributed to early diagnosis^6^.

According to a study investigating the epidemiology of cervical cancer in Central Asian countries (Tajikistan, Turkmenistan, Uzbekistan, Kyrgyzstan, and Kazakhstan) for the year 2018, the combined estimates for all five countries showed an incidence rate of 12.5 and a mortality rate of 12.3 per 100,000 women. According to the data provided by the authors, the incidence and mortality rates in Tajikistan were 5.7 and 3.2 per 100,000 women, respectively and in Turkmenistan, 13.6 and 8.8 per 100,000 women. Also, based on the results of this study in Uzbekistan, incidence and mortality rates from cervical cancer were 9.9 and 5.4 per 100,000 women, respectively and in Kyrgyzstan 19.9 and 10.9 per 100,000 women. In Kazakhstan the incidence rate was 15.7 and the mortality rate 7.5 per 100,000 women^27^.

There’s a notable disparity between our findings and those of a previous study on cervical cancer in Central Asia^27^, which highlighted variations in incidence and mortality rates among the region’s countries. We suggest this difference might be due to the timing of national cervical cancer screening program implementations. Countries with higher mortality rates, like Kazakhstan and Turkmenistan, initiated screening earlier (2008 and 2007 respectively), whereas in Kyrgyzstan, where rates were also high, cytological screening started in 2013 without a clear indication of existence a national screening program^27^. This trend likely reflects active screening efforts, expanding the pool of examined women and consequently enhancing cervical cancer case detection.

When comparing Central Asian countries with high-income nations, it becomes evident that, incidence and mortality rates are substantially higher in Central Asian countries. Specifically, in the United States and Canada, the incidence of cervical cancer is 6.5 and 5.7 per 100,000 women, respectively, and the mortality rates are 1.9 and 3.1 per 100,000 women, respectively. Switzerland’s incidence of cervical cancer is 3.8, with a mortality rate of 1.1 per 100,000 women, while in Finland, the incidence is 4.7 and the mortality rate is 0.9 per 100,000 women^27^.

A 2012 study carried out in China revealed that the incidence and mortality rates of cervical cancer in China are not evenly distributed^28^, with significant disparities observed between rural and urban areas, as well as the eastern, central, and western regions of the country^29^. According to a study carried out in Romania, there has been a notable decrease in mortality rates from cervical cancer, with a reduction of 13% observed in 2019 in comparison to 2001. A significant decrease in mortality was registered in the rural areas of Romania (20% compared to 5% in urban areas)^30^.

A further previous study conducted in Kazakhstan highlights geographical disparities in the incidence of cervical cancer. According to the authors, low incidence rates (per 100,000 female population) were observed in the southern regions of the country, specifically Southern Kazakhstan (12.3), Kyzylorda (13.0), Mangistau (13.3), and Zhambyl (13.9). The regions of Almaty, Karaganda, Atyrau, and Akmola ranked average in terms of the incidence of cervical cancer, while East Kazakhstan emerged as the region with the highest incidence rate^26^. These disparities, same as in our study, could potentially be attributed to variations in the quality of the implementation of the cervical cancer screening program across different regions, with active screening potentially leading to higher incidence rates. Additionally, demographic factors such as migration might also contribute to these differences.

Prior studies suggest that regional disparities in CC incidence may relate to demographic, urbanization, and medical factors impacting risk and prognosis of CC. In one study, high mortality rates in western regions were linked to climate and environmental issues. But it is worth noting that cervical cancer screening in all regions of Kazakhstan is fully available to the insured population and it is planned to conduct screening according to the guaranteed volume of free medical care^31–33^.

### Study on attitude to cervical cancer screening in urban and rural areas

In this part of our study our findings showed significant differences between the two groups regarding participation rates, regularity of visits, dates of last screening visit, and awareness of cervical cancer screening. Specifically, rural women exhibited lower commitment to screening, attending less frequently and with less regularity, and having lower awareness of the screening program compared to urban women. Nonetheless, a majority of women living in rural areas expressed an intention to undergo cervical cancer screening and believed that regular notifications about upcoming screenings were necessary.

Researchers found that women residing in rural areas and engaged in household occupations were more likely to decline cervical cancer screening. Additionally, most respondents did not perceive themselves to be at risk of cervical cancer (83.1%). Lack of time, confidence in their health, and fear of screening were the primary reasons cited for declining screening. Perceived time constraints and a lack of perceived risk were identified as the primary obstacles to screening. Moreover, residing in rural areas and engaging in household activities were associated with lower screening participation rates^34^. Other research shows that the main obstacle is the prohibitive cost of medical services and lack of health insurance coverage in countries without government programs^35–37^. In developing countries, limited infrastructure and a shortage of qualified medical personnel, socio-cultural factors such as limited awareness, fear of screening and positive test results, social stigma, embarrassment, and concerns about confidentiality also significantly barriers to participation in screening programs^37–39^.

Earlier studies in Kazakhstan identify significant barriers to timely cervical cancer screening, including insufficient medical knowledge, low commitment, distrust in the need for screening, lack of information about its availability and effectiveness, time constraints, fear of a cancer diagnosis, and inadequate understanding of the procedure’s importance^40,41^.

Our research results also revealed low screening participation rates among rural women. Although rural women overwhelmingly recognized the importance of timely diagnosis of cervical cancer, we did not investigate the underlying reasons for not undergoing screening or analyze the employment status of rural women. Foreign researchers have reported similarly low levels of awareness of cervical cancer screening among rural women^42^. One study examining the sociological and anthropological perspectives on rural women’s perceptions and knowledge of cervical cancer screening and symptoms found that 95.78% of women surveyed had never undergone screening and had limited understanding of the disease in terms of its causes, prevention, and treatment^43^.

Our study has limitations that must be considered. A limitation of the study of trends is the different time period, which does not allow to fully describe the situation of epidemiological indicators of cervical cancer in the Republic of Kazakhstan.

One potential limitation of the study is the snowball sampling, which may not yield a fully representative sample due to participants’ tendency to recruit individuals similar to themselves. Consequently, the employment of snowball sampling may introduce selection bias. Additionally, since this study served as a pilot, it is important to note its limitations, including a constrained sample size.

The use of an electronic questionnaire may have led to respondents providing less than truthful answers and can be a bias in selecting cases from rural areas, which could have influenced the results. Moreover, we did not examine the reasons for non-participation in screening or conduct an analysis of potential disparities in access to cervical cancer screening between rural and urban women. A systematic review on cervical cancer screening in rural areas highlighted several studies that have underscored the persistent problem of unequal access to diagnostic and treatment services for cervical cancer between rural and urban areas within countries^44,45^.

Our study did not examine environmental factors, healthcare access, genetic predispositions, or cultural practices and their influence on differences between regional cervical cancer incidence rates and what is needed when studying cervical cancer. These limitations emphasize the need for further research to address these issues in future studies.

### Implications for policy makers

To effectively curb the incidence and mortality rates of cervical cancer, it is imperative to adopt policy strategies that prioritize both prevention and treatment. Among the most successful preventive strategies is vaccination, particularly through the use of the HPV vaccine, which has been proven to significantly reduce the incidence of HPV strains that are most commonly linked to cervical cancer^46^. As such, policies geared towards increasing access to HPV vaccination must be given high priority. This can be achieved through initiatives such as providing low-cost or free vaccinations to girls and young women and implementing vaccination programs in schools and healthcare facilities. In Kazakhstan, for instance, plans to integrate HPV vaccination into the national vaccination schedule have been made, although a pilot vaccination program failed due to high levels of vaccine hesitancy^47^. However, despite the negative outcome of the pilot program, HPV vaccination should be implemented into the national vaccination schedule accompanied by a huge information campaign, especially for parents, adolescents and teachers.

Increasing access to screening tests is another crucial prevention strategy for reducing cervical cancer mortality rates. Regular screening can detect pre-cancerous changes in the cervix and enable early intervention to prevent progression to cancer. In this instance, it is plausible that the incidence rate may rise as a result of expanded testing coverage, nevertheless, mortality rates of cervical cancer will exhibit a decline, because cases will be treated earlier. Policies should prioritize making screening tests widely available, especially in underserved communities. This could entail providing subsidies for screening tests, mobile screening clinics, and awareness campaigns that emphasize the importance of regular screening. Despite the fact that cervical cancer screening has been implemented in Kazakhstan since 2008, the uptake of the screening program in Kazakhstan remains suboptimal, indicating the need for awareness-raising campaigns to improve participation rates.

Early detection plays a crucial role in the successful treatment of cervical cancer. Hence, policies should prioritize improving access to treatment options. This entails ensuring that women have access to effective therapies, including surgery, radiation therapy, and chemotherapy. In addition, policies should strive to reduce the financial burden of treatment by offering subsidies or insurance coverage for cancer care. Given that oncology care is a key priority for the healthcare system in Kazakhstan, the Ministry of Health (MoH) has planned and implemented a series of health plans that aim to provide state-of-the-art therapy to cancer patients. Nevertheless, efforts to secure funds for an improved treatment of cervical cancer patients should be maintained.

Education plays a crucial role in the prevention and control of cervical cancer. Policy makers should prioritize increasing awareness of the risk factors associated with cervical cancer, including smoking, human papillomavirus (HPV) infection, and compromised immune systems. Additionally, educational programs can be implemented to promote healthy behaviors that decrease the likelihood of developing cervical cancer, such as safe sexual practices and regular screenings. Lastly, policies should address the social and economic factors that contribute to cervical cancer incidence and mortality, which may involve enhancing access to healthcare services in underserved communities, providing support for low-income families, and tackling gender inequalities that restrict women’s access to healthcare and education.

## Conclusion

The results of the trend study of CC conducted between 2013 and 2019 in the Republic of Kazakhstan indicate that, despite a decrease in mortality rates, the incidence of cervical cancer has remained high and stable.

Furthermore, the study comparing the attitudes of urban and rural women towards cervical cancer screening highlighted significant differences in screening participation rates, frequency of visits, and awareness among the target age group. Women living in rural areas, less committed to screening due to lower program awareness, highlight the need to increase awareness and incentivize screening across all target groups. Effective interventions are essential in both urban and rural areas to raise awareness and participation in cervical cancer screening, aiming to reduce incidence and enhance women’s health in Republic of Kazakhstan.

## Data Availability

The datasets used and analysed during the current study are available from the corresponding author on reasonable request.

## Abbreviations

CC: Cervical cancer
HPV: Human papillomavirus
IARC: International Agency for Cancer Research
WHO: World Health Organization
KCR: Kazakhstan Cancer Registry
KazIOR: Kazakh Institute of Oncology and Radiology
ESP: European Standard Population
WSP: World Standard Population
CI: Confidence interval
WinPepi: Programs for epidemiologists in windows
SPSS: Statistical Package for the Social Sciences
MoH: Ministry of Health
